# Editorial: Spotlight on aging: role of exercise and nutrition in healthy longevity

**DOI:** 10.3389/fragi.2025.1698219

**Published:** 2025-10-09

**Authors:** Antonio Paoli, Richard Siow, Tatiana Moro

**Affiliations:** ^1^ Department of Biomedical Sciences, University of Padova, Padova, Italy; ^2^ School of Cardiovascular and Metabolic Medicine and Sciences, Faculty of Life Sciences and Medicine, and Ageing Research at King’s, King’s College London, London, United Kingdom; ^3^ Department of Physiology, Anatomy and Genetics, University of Oxford, Oxford, United Kingdom

**Keywords:** resistance trainig, microbiome, miRNA, endurance exercise, physical exercise, mind - body approaches, aging, healthy ageing

The demographic shift toward an increasingly older population presents one of the most significant challenges for global health systems. While life expectancy has improved dramatically over the past century, recent decades have seen a slowing of these gains (Olshansky et al., 2024). Moreover, additional years are not always matched by equivalent gains in healthspan, the portion of life spent in good health, free from chronic disease and disability. The dual goals of extending life and optimizing its quality have propelled research into modifiable lifestyle factors, particularly exercise and nutrition, as central pillars of healthy aging (Woods et al., 2024).

A growing body of evidence indicates that physical activity and diet interact in complex, synergistic ways to modulate the biological processes underlying aging. Nutritional strategies such as adherence to a Mediterranean-style diet have been associated with reduced inflammation, improved metabolic control, and lower incidence of frailty (Roman et al., 2008; Ros et al., 2014). In parallel, both the total amount of physical activity and structured exercise programs exert potent effects on cardiovascular (showing a moderate to large effect) (Huang et al., 2005), neuromuscular (large effect) (Eidam et al., 2024), and cognitive function (large effects) (Concannon et al., 2012; Moro and Paoli, 2020; Han et al., 2025). It is important to note that, although related, they are not synonymous: physical activity (PA) refers to any movement produced by the musculoskeletal system that increases energy expenditure above rest, whereas physical exercise (PE) is a planned, structured, and purposeful form of PA (Paoli, 2025).

In this Research Topic, authors explored the effects of both resistance training (RT) and endurance training (ET), emphasizing that these two exercise types share some benefits but also exert distinct effects through different molecular and metabolic pathways. Together, they influence epigenetic regulation, mitochondrial health, and systemic inflammation, shaping the trajectory of aging at the molecular, organ, and whole-body levels.

The first contribution, Cione et al., underscores the importance of RT in combating age-associated muscle loss. The downstream pathways of different kinds of exercise have been largely investigated in their mainly components: the mTOR/protein translation in resistance exercise vs. PGC1α/oxidative metabolism in endurance exercise (Smith et al., 2023). Focusing on the less investigate role of muscle-specific microRNAs (myomiRs) in sarcopenia, the authors highlight emerging molecular biomarkers that could help tailor exercise and nutrition interventions. This mechanistic perspective is particularly relevant given that both exercise (in this case RT) and diet can modulate microRNA expression, with downstream effects on muscle protein synthesis and regenerative capacity (Margolis and Rivas, 2018).

The second article: a randomized controlled trial protocol, Merelim et al., investigates how different exercise modalities influence the gut microbiome in sarcopenic older adults, a rapidly expanding area of research. While early studies highlighted the strong impact of nutrition on gut microbial composition, only in recent years has the role of exercise been recognized (Mancin et al., 2021; Quaresma et al., 2024), not only on performance but also on exercise motivation (Dohnalova et al., 2022). To date, limited data exist on how different types of exercise affect the microbiota in older adults (Lavilla-Lerma et al., 2024). In this trial, the authors compared high-intensity interval training (HIIT) and moderate-intensity continuous training (MICT) in their effects on gut microbiota composition, muscle mass, and physical performance. Preliminary results show that both interventions were well tolerated and improved muscle strength and gait speed, with HIIT producing greater gains in lower-limb power. Microbiome analyses revealed increases in α-diversity and enrichment of short-chain fatty acid–producing taxa (e.g., *Faecalibacterium prausnitzii*) in the HIIT group, while MICT was associated with stabilization of diversity and modest increases in *Bifidobacterium*.

These findings confirm that exercise modality can differentially modulate both muscle function and gut microbial ecology in aging. Moreover, variations in exercise intensity during cyclic movements such as running or cycling may influence the adaptive response in older adults. Investigating this relationship was the aim of the review by Zolla and colleagues Zoila et al., who analyzed the effects of high-intensity interval training (HIIT) versus continuous aerobic training in this population. They found that HIIT elicited greater improvements in VO_2_max, balance, and executive function, whereas continuous training was more effective for enhancing mood and supporting adherence. These complementary outcomes align with meta-analytic evidence indicating that both approaches confer benefits for physical (Oliveira et al., 2024) and cognitive health (Tsai et al., 2021). Ancient mind–body practices can also yield significant benefits for older adults. In this *Research Topic*, Kaushik et al. explored the effects of a 12-week program that integrated physical postures, breathing techniques, and meditation for people living with Alzheimer’s disease and their primary caregivers. Participants with Alzheimer’s showed measurable gains in cognitive performance on the Mini-Mental State Examination, along with reductions in depressive mood, perceived stress, and greater independence in daily tasks. Caregivers experienced improved quality of life and a lighter sense of burden, suggesting yoga as a practical, affordable approach with benefits that extend to both patients and their care partners. Building on the evidence from yoga, other gentle mind–body activities can also be valuable for conditions associated with aging. Indeed, Author et al., 2025 in their review examined the evidences about the effects of Tai Chi—an ancient Chinese martial art—alongside various forms of physical activity and exercise (including walking, cycling, swimming, aquatic training, and resistance work) on outcomes in knee osteoarthritis. Tai Chi was found to provide meaningful improvements in pain management (improvement of pain via the WOMAC scale by 66–755 over 12 weeks), postural stability, and functional mobility. Similarly, land-based strengthening and aerobic programs performed two to three times per week over 8–12 weeks produced notable gains in pain reduction, physical function, and quality of life. Aquatic exercise emerged as a helpful option for individuals with greater mobility limitations, while moderate-intensity training with progressive loading appeared optimal for many. The authors concluded that all exercise types examined offered comparable benefits for knee osteoarthritis, with no single “best” modality identified. This aligns with findings from a randomized controlled trial in 80 retirement-village residents, which showed that overall physical activity levels—rather than specific exercise types or intensities—were most strongly linked to better vascular health, as assessed by pulse wave velocity Hill et al.. These results reinforce the message that, for older adults, simply being active on a regular basis may be more important than the exact form the activity takes (Gomez-Sanchez et al., 2023). From resistance training and endurance exercise to mind-body physical activities, this Research Topic reinforces the broad benefits of being active in later life, especially when combined with optimal nutrition. They illustrate that while specific modalities may target particular outcomes, the overarching message is clear: movement, in its many forms, is a cornerstone of healthy aging.
